# Clinical case series: Emergence of psoriatic arthritis during biologic IL-23 pathway inhibition therapy for psoriasis

**DOI:** 10.3389/fmed.2026.1962420

**Published:** 2026-09-30

**Authors:** Andrew Z. Li, Ian D. Cooley, Ethan Craig, Chris T. Derk, Shubhasree Banerjee, Alexis R. Ogdie, Kelcie L. Molchany, Joel M. Gelfand

**Affiliations:** 1 Department of Dermatology, Perelman School of Medicine, University of Pennsylvania, Philadelphia, PA, United States; 2 Department of Rheumatology, Perelman School of Medicine, University of Pennsylvania, Philadelphia, PA, United States, Philadelphia, PA, United States; 3 Nesbitt School of Pharmacy, Wilkes University, Wilkes-Barre, PA, United States

**Keywords:** dermatology, guselkumab, IL-12/23 inhibitors, IL-23 inhibitors, psoriasis, psoriatic arthritis, risankizumab, ustekinumab

## Abstract

Psoriasis (PsO) and psoriatic arthritis (PsA) are increasingly recognized as manifestations within the spectrum of psoriatic disease, and emerging observational data suggest that biologic therapy targeting interleukin-23 (IL-23) may reduce the risk of incident PsA. This finding remains controversial given the potential for channeling and information bias. Here, we describe five patients with long-standing PsO who developed new-onset PsA while achieving excellent skin clearance with IL-23 or IL-12/23 inhibitor therapy (risankizumab in four patients and ustekinumab in one). The time from the initiation of IL-23 pathway inhibition to PsA diagnosis ranged from 5 to 63 months. All patients met the ClASsification criteria for Psoriatic ARthritis, with musculoskeletal manifestations including dactylitis, synovitis, tenosynovitis, and enthesitis, and two patients had erosive changes on imaging. Joint symptoms improved in the majority of patients after transition to an IL-17-targeted biologic. These cases indicate that IL-23 pathway inhibition may not reliably prevent the onset of PsA and may contribute to its development in susceptible individuals. Thus, this series underscores the importance of ongoing musculoskeletal surveillance in patients with PsO receiving IL-23-targeted therapy, even when skin disease is completely controlled.

## Introduction

Psoriasis (PsO) is a chronic, immune-mediated inflammatory skin disorder associated with psoriatic arthritis (PsA), cardiometabolic disease, and premature cardiovascular mortality (1). PsA typically occurs on or after the onset of PsO and affects approximately 10%–30% of patients, with higher prevalence among those with nail, scalp, palmoplantar, or genital involvement, and patients with obesity, a family history of PsA/PsO, and more extensive skin disease (2–4). Emerging observational studies suggest that biologic treatments, particularly those targeting interleukin-23 (IL-23), may prevent PsA development (5, 6). This finding is controversial because there is strong channeling bias and information bias (e.g., how patients are coded) that could impact the results (7). Here, we report a case series of patients who developed inflammatory arthritis while being successfully treated for PsO with IL-23-targeting biologics. Without a comparator group, this case series cannot establish whether IL-23 inhibition failed to prevent or paradoxically contributed to PsA onset in these patients. Rather, we aim to characterize the clinical course of incident PsA occurring during IL-23 inhibitor therapy and to emphasize the need for vigilant musculoskeletal monitoring in this setting. PsA diagnoses were confirmed by rheumatologists using the Classification criteria for Psoriatic Arthritis (CASPAR) and imaging when indicated.

## Case descriptions

### Patient 1

A 31-year-old man with an 18-year history of scalp PsO, no comorbidities or history of inflammatory bowel disease (IBD) and uveitis, and a family history of PsO in his paternal grandmother presented after loss of response to 10 years of treatment with adalimumab. At presentation, his BMI was 24 kg/m^2,^ and he had 3% body surface area (BSA) involvement, with a physician global assessment (PGA) of 3 (moderate). He was transitioned to risankizumab, an IL-23 inhibitor, achieving complete skin clearance.

After 15 months on risankizumab, he developed morning stiffness and joint pain in the metatarsophalangeal (MTP) and right metacarpal (MCP) joints. The examination was unremarkable, but the symptoms persisted. At 22 months, a rheumatology evaluation revealed dactylitis of the left fourth toe and right ankle tenosynovitis confirmed by ultrasound (Figure 1). No enthesitis was reported.

**Figure 1:**
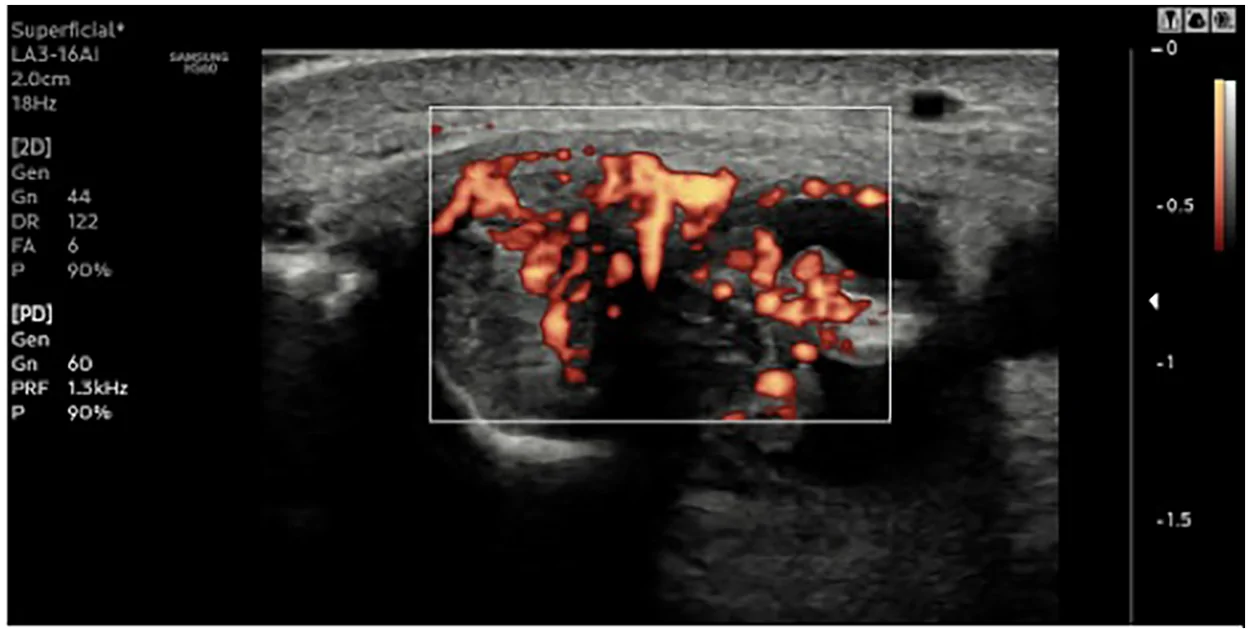
Ultrasound of the posterior tibial and flexor digitorum tendons (transverse view at the level of the malleolus) indicating highly active posterior tibial and flexor digitorum longus tendinitis and tenosynovitis.

Laboratory studies were negative for rheumatoid factor (RF) and cyclic citrullinated peptide (CCP), with an erythrocyte sedimentation rate (ESR) of 27, C-reactive protein (CRP) of 1.5 mg/dL, and uric acid of 6.0 mg/dL. He met the CASPAR criteria and was diagnosed with PsA. Risankizumab was discontinued and replaced with bimekizumab, an IL-17A/F inhibitor, resulting in an improvement in joint symptoms and sustained skin clearance, although mild residual joint inflammation persisted.

PsA was diagnosed 22 months after risankizumab initiation, despite the patient having completely clear skin.

### Patient 2

A 27-year-old woman with a 17-year history of PsO, no comorbidities or history of IBD or uveitis, and no family history of PsO or PsA presented after failing topical corticosteroids and phototherapy. At presentation, her scalp was the predominant location [4% BSA, PGA of 3 (moderate)], and her BMI was 23 kg/m^2^. Risankizumab was started, and she achieved complete skin clearance.

After 21 months on risankizumab, she developed right second proximal interphalangeal (PIP) joint tenderness, initially attributed to repetitive strain. Three months later, she developed dactylitis of the right second digit and morning stiffness of the temporomandibular joint (TMJ). A rheumatologic evaluation confirmed synovitis without enthesitis; laboratory studies reported a CRP of 1.7 mg/dL, and she was confirmed to meet the CASPAR criteria. A head/neck MRI was conducted for TMJ symptoms only and not to assess for axial disease, given the lack of axial symptoms, and demonstrated a small TMJ effusion and possible disc degeneration.

She was diagnosed with PsA and transitioned to secukinumab, an IL-17A inhibitor, at 300 mg every 4 weeks after experiencing continued joint symptoms and dactylitis of the right third toe. Secukinumab resulted in a 75% self-reported improvement in joint symptoms and sustained skin clearance. However, she developed persistent synovitis and tenosynovitis, confirmed clinically and by ultrasound (Figure 2). Apremilast was added but discontinued due to gastrointestinal side effects despite clinical benefit.

**Figure 2:**
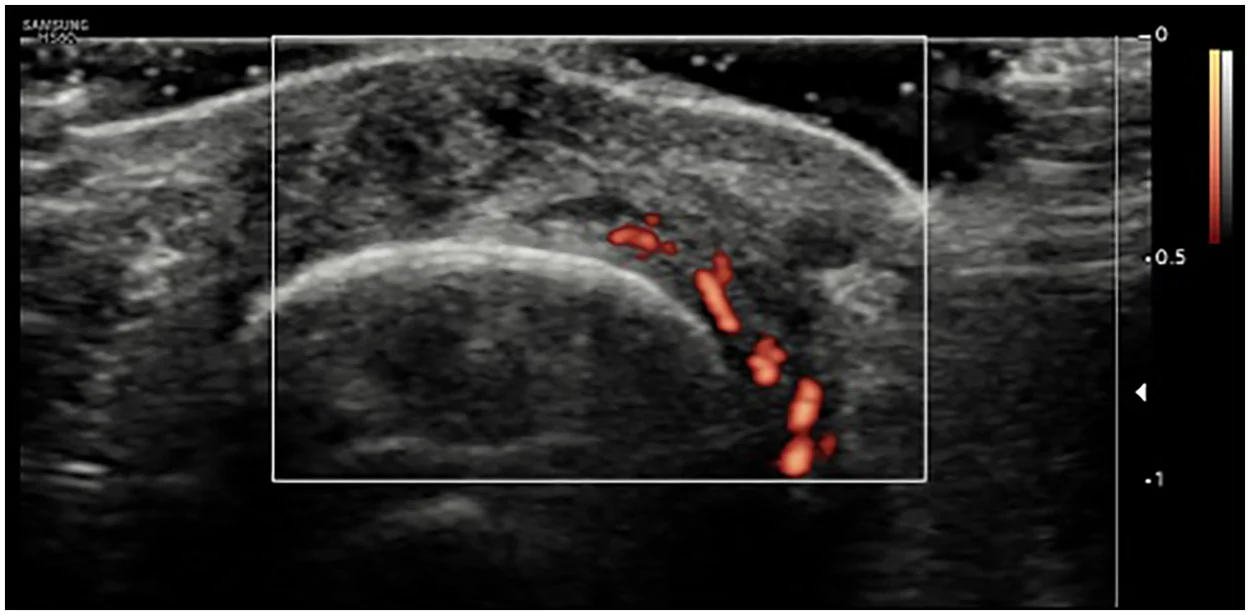
Ultrasound of the second right MCP (dorsal transverse view) indicating synovitis and tenosynovitis.

After approximately 10 months on secukinumab, she was subsequently switched to bimekizumab, an IL-17A/F inhibitor, leading to improvement with minimal residual tenderness and no active synovitis. Approximately 1 year later, she developed worsening stiffness and pain in the right second MCP joint. Sulfasalazine was initiated and titrated to 3 g/day, with modest improvement. X-rays showed no evidence of joint space narrowing or soft tissue swelling. Due to continued symptoms in the right second MCP joint, she was subsequently transitioned from bimekizumab to adalimumab, a tumor necrosis factor (TNF)-alpha inhibitor, resulting in improvement of joint symptoms with sustained skin clearance.

PsA was diagnosed 24 months after risankizumab initiation despite clear skin.

### Patient 3

A 40-year-old woman with a 10-year history of PsO, a past medical history of juvenile rheumatoid arthritis (JIA) in childhood, obesity, a family history of PsO and PsA in her maternal grandmother and sister, and without a history of IBD and uveitis, presented after loss of response to etanercept. At presentation, her BMI was 31 kg/m^2,^ and she had 5%–10% BSA, with significant involvement of her plantar feet and nails. At this initial visit, she was put on topical PUVA (Psoralen + UVA light). Three years later, her PsO had progressed to involve 15% BSA and a PGA of 3 (moderate). She was then transitioned to ustekinumab, an IL-12 and IL-23 inhibitor, achieving near-complete clearance (<1% BSA and a PGA of 1).

Four years later, she developed dactylitis and joint pain involving her hands, knees, and neck, despite minimal skin activity. Dose escalation of ustekinumab did not improve symptoms. A rheumatologic evaluation revealed ankle synovitis, nail dystrophy, dactylitis, and joint tenderness, all without enthesitis. She met the CASPAR criteria with negative RF and CCP, ESR of 6, CRP of 3.9 mg/dL, and unremarkable hand and knee imaging. No cervical spine or other axial imaging was conducted, and HLA-B27 was not determined. Given these laboratory findings, her personal and family history of PsO, psoriatic domains of joint involvement, and no recurrent JIA symptoms since childhood, new-onset PsA was favored over recurrence of JIA. She was diagnosed with PsA and transitioned to ixekizumab, an IL-17A inhibitor, resulting in significant improvement in joint symptoms and complete skin clearance.

Over the next few years, she experienced intermittent joint symptoms with persistent dactylitis and fluctuating inflammatory markers with clear skin.

PsA was diagnosed 63 months after starting ustekinumab, despite clear skin.

### Patient 4

A 67-year-old woman with a 37-year history of PsO, a past medical history of Lichen sclerosus, immune thrombocytopenic purpura status post splenectomy, atrial fibrillation, a family history of PsO and PsA in her mother and daughter, and without a history of IBD and uveitis, presented after failing topical corticosteroids and phototherapy. At presentation, her BMI was 35 kg/m^2,^ and she had >10% BSA and a PGA of 3 (moderate). She was then started on phototherapy and tapinarof cream for 4 months with poor response and was subsequently transitioned to risankizumab.

After 4 months, she achieved complete clearance, but she developed worsening joint pain involving her hands, knees, and hips. A rheumatologic evaluation showed tenderness in multiple PIP joints, without enthesitis. She met the CASPAR criteria, and her laboratory studies reported an ESR of 62, a CRP of 0.9 mg/dL, a uric acid of 6.6 mg/dL, negative RF and CCP, and negative antinuclear antibodies. X-rays showed no erosions, but ultrasound demonstrated synovial hypertrophy and thickening and microerosions consistent with inflammatory arthritis. She was diagnosed with PsA, and it was recommended that she change to an IL-17-targeting biologic, but she elected to continue risankizumab.

PsA was diagnosed 5 months after risankizumab initiation despite complete skin clearance.

### Patient 5

A 45-year-old man with a 9-year history of PsO, a past medical history of metabolic syndrome, mixed hyperlipidemia, low-level monoclonal gammopathy of undetermined significance, a family history of PsO in his half-brother, and without a history of IBD and uveitis, presented after poor response to topical corticosteroids. At presentation, his BMI was 30 kg/m^2,^ and he had 7% BSA and a PGA of 3 (moderate). He was then started on adalimumab. After 2 years, he continued to have a poor response to treatment, with 3% BSA and a PGA of 3 (moderate). He was then transitioned to risankizumab and achieved complete clearance.

Approximately 12 months later, he developed swelling and pain in the toes and fingers. A rheumatologic evaluation confirmed PsA after meeting the CASPAR criteria, with a CRP of 0.9 mg/dL, a uric acid of 4.9 mg/dL, and negative RF and CCP. Ultrasound demonstrated MCP and MTP synovitis and erosive changes, flexor pollicis tenosynovitis and enthesitis, and left extensor hallucis enthesitis (Figure 3).

**Figure 3:**
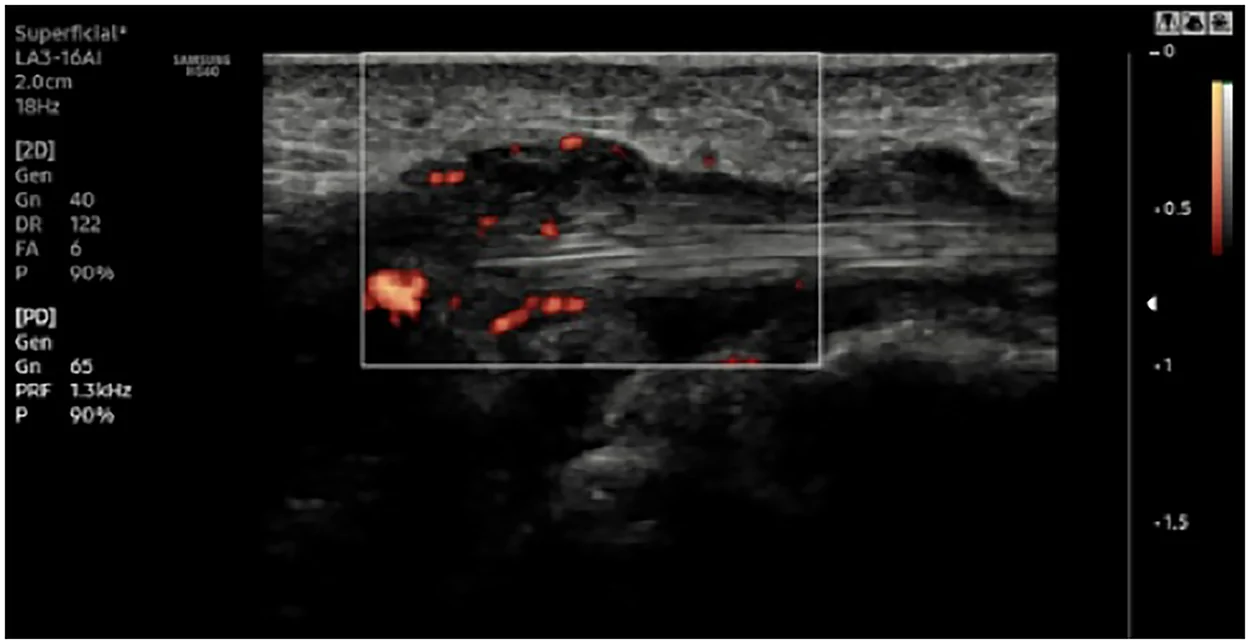
Ultrasound of the left flexor pollicis longus (volar longitudinal view) indicating flexor pollicis tenosynovitis and enthesitis.

He later developed mild recurrence of PsO and was transitioned to ixekizumab, an IL-17A inhibitor, which improved his joint symptoms but did not fully restore skin control, resulting in 1% BSA and a PGA of 3 (moderate).

PsA was diagnosed 44 months after risankizumab initiation, despite prior clear skin.

## Discussion

PsO and PsA are increasingly recognized as manifestations within the spectrum of psoriatic disease. The development of PsA during treatment with IL-23 or IL-12/23 inhibitors has not been well described, but isolated case reports describe new-onset or paradoxical PsA arising during guselkumab and ustekinumab therapy despite an adequate skin response (8, 9). These reports, together with our series, contrast with observational data suggesting that IL-23 and IL-17 inhibitors are associated with a lower incidence of incident PsA than TNF inhibitors (5, 6). However, the observed protective effect of IL-23 biologics on the development of PsA may be spurious (e.g., due to channeling and collider bias), and ultimately, randomized controlled trials are needed to test this hypothesis (10). The temporal relationship in our five cases demonstrates that PsA can still develop in patients on IL-23 biologics, despite excellent skin control (Table 1).

**Table 1:** Results of biologic IL-23 pathway inhibition treatment on psoriasis severity and PsA diagnosis.

Patient ID	Sex	Age	Family history of PsO and/or PsA	Prior treatments for psoriasis	Comorbidities	Duration of psoriasis (years)	IL-23 or IL-12/23 inhibitor treatment	Psoriasis severity immediately prior to IL-23/IL-12-23 inhibitor treatment	Psoriasis severity during treatment	Time to PsA diagnosis	Imaging findings	Switched medication that improved joint symptoms
1	M	31	PsO (paternal grandmother)	Adalimumab	None	18	Risankizumab	BSA 3%, PASI 8	BSA 0%, PASI 0	22 months	Ankle US: tenosynovitis	Bimekizumab (IL-17 inhibitor)
2	F	27	None	Topical corticosteroids, phototherapy	None	17	Risankizumab	BSA 3%, PASI 9	BSA 0%, PASI 0	24 months	Head/neck MRI: TMJ effusion, possible disc degeneration; Hand US: MCP synovitis/tenosynovitis; hand X-ray: unremarkable	Secukinumab, bimekizumab (IL-17 inhibitors), then adalimumab (TNF-alpha inhibitor)
3	F	40	PsO (maternal grandmother, sister); PsA (sister)	Etanercept, PUVA	Obesity	10	Ustekinumab	BSA 15%, PGA 3	BSA 0%, PASI 0	63 months	Hand X-ray: unremarkable	Ixekizumab (IL-17 inhibitor)
4	F	67	PsO (mother, daughter); PsA (daughter)	Triamcinolone, phototherapy, tapinarof	Lichen sclerosus, ITP s/p splenectomy, atrial fibrillation	37	Risankizumab	BSA >10%, PGA 3	BSA 0%, PGA 0	5 months	Hand US: MCP synovial hypertrophy, thickening, microerosions	No change (continued risankizumab)
5	M	45	PsO (half-brother)	Topicals, adalimumab	Metabolic syndrome, hyperlipidemia, MGUS	9	Risankizumab	BSA 3%, PGA 3	BSA 0%, PGA 0; later BSA 1%, PGA 2-3	44 months	Hand US: synovitis, tenosynovitis, enthesitis, erosive changes	Ixekizumab (IL-17 inhibitor)

PsO, psoriasis; PsA, psoriatic arthritis; BSA, body surface area; PGA, physician global assessment; PASI, psoriasis area and severity index; MCP, metacarpophalangeal; ITP, immune thrombocytopenia; MGUS, monoclonal gammopathy of undetermined significance; TMJ, temporomandibular joint; US, ultrasound.

The following three possibilities could explain these cases: IL-23 blockade may truly contribute to PsA development; it may unmask previously subclinical PsA in susceptible individuals; or PsA may arise through IL-23-independent inflammatory pathways that are inadequately suppressed by IL-23 inhibition. Our case series, which lacks a comparator group, cannot distinguish among these possibilities. Notably, three of the five patients (Patients 1, 3, and 5) had previously failed anti-TNF therapy before starting an IL-23 inhibitor. Patients with prior biologic exposure and treatment-refractory disease may constitute a subgroup with a different baseline risk of PsA.

IL-23 is a key driver of pathogenic Th17 differentiation and IL-17 production, so its inhibition would be expected to reduce IL-17-mediated inflammation. However, IL-17 production in PsA is not exclusively IL-23-dependent. γδ T cells, mucosal-associated invariant T cells, and other innate-like lymphocytes can generate IL-17A/F independently of IL-23 (11). Stober et al. found that polyfunctional Th17 and Th17-derived cells were enriched directly within the inflamed joints of patients with PsA, and separately showed, using *in vitro* differentiation of patient-derived CD4 T cells, that withholding IL-23 reduced but did not completely prevent IL-17 production, demonstrating that IL-17 release can occur through IL-23-independent pathways (12). This mechanism could explain persistent or new joint inflammation despite adequate IL-23 blockade and is consistent with the improvement that several of our patients experienced after switching to an IL-17 targeted biologic. However, this does not prove that IL-23 inhibition caused the arthritis, since IL-17 inhibitors are independently effective in treating PsA.

A related mechanism is the compensatory upregulation of alternative cytokine pathways following targeted cytokine blockade, or “cytokine escape.” TNF blockade can increase Th17/IL-17 responses (13), IL-4/IL-13 blockade has been associated with increased Th17-mediated inflammation (14), and IL-17 blockade may promote TNF- and type I interferon-driven pathways (15). A similar compensatory cytokine shift could theoretically result from IL-23 inhibition and contribute to musculoskeletal inflammation despite skin clearance. This concept has resulted in growing interest in combining biologic therapies for patients with PsO with recalcitrant disease (16).

A related question is whether these presentations reflect a phenotypic shift analogous to the pustular, follicular, or flexural flares well documented in paradoxical PsO during biologic therapy. Notably, none of our patients demonstrated this pattern. All of the patients achieved and largely maintained complete or near-complete skin clearance without developing a new PsO morphology. In addition, there was not an obvious transition from axial to peripheral joint disease, as all five presentations were peripheral (dactylitis, synovitis, tenosynovitis, and enthesitis) from onset.

## Conclusion

This case series highlights five patients who developed PsA following treatment with IL-23 pathway inhibitors for PsO. PsA proved difficult to control in several of these patients, and two developed erosions on imaging. While IL-23 inhibitors have demonstrated efficacy in treating PsO and PsA in previous studies, these cases suggest that IL-23 inhibition may not prevent the development of PsA and may possibly contribute to its onset in susceptible individuals. Since this is a case series without a comparator group, these observations are hypothesis-driven rather than causal, and subclinical axial involvement cannot be excluded given that axial imaging was not performed on many of these patients. Further research is needed to understand the mechanisms behind these findings and to identify predictive factors for PsA development in patients with PsO receiving treatments that inhibit IL-23. It is important for physicians to screen for musculoskeletal symptoms in patients with PsO on these treatments, even when their skin is completely clear, and to consider alternative biologic therapies, particularly IL-17 inhibitors, if PsA develops.

## Patient perspective

The joint symptoms seen in these five patients had a large impact on their daily lives, affecting their work and home life. These symptoms were severe enough for them to seek care. After changing biologics, the patients reported a noticeable improvement in their joint symptoms.

## Data Availability

The data analyzed in this study are subject to the following licenses/restrictions: Private patient data from EMR data. Requests to access these datasets should be directed to Joel.Gelfand@pennmedicine.upenn.edu.
